# Identification of Genetic Features for Attenuation of Two *Salmonella* Enteritidis Vaccine Strains and Differentiation of These From Wildtype Isolates Using Whole Genome Sequencing

**DOI:** 10.3389/fvets.2019.00447

**Published:** 2019-12-18

**Authors:** Yue Tang, Rob Davies, Liljana Petrovska

**Affiliations:** Department of Bacteriology, Animal and Plant Health Agency, Addlestone, United Kingdom

**Keywords:** *Salmonella* Enteritidis, vaccine, whole genome sequencing, differentiation, characterization

## Abstract

*Salmonella* Enteritidis is a major cause of salmonellosis worldwide and more than 80% of outbreaks investigated in Europe have been associated with the consumption of poorly cooked eggs or foods containing raw eggs. Vaccination has been proven to be one of the most important measures to control *Salmonella* Enteritidis infections in poultry farms as it can decrease colonization of the reproductive organs and intestinal tract of laying hens, thereby reducing egg contamination. Differentiation of live vaccine from field or wild type *S*. Enteritidis isolates in poultry is essential for monitoring of veterinary isolates and targetting control actions. Due to decreasing costs, whole genome sequencing (WGS) is becoming a key tool for characterization of *Salmonella* isolates, including vaccine strains. Using WGS we described the genetic changes in the live attenuated Salmovac 440 and AviPro SALMONELLA VAC E vaccine strains and developed a method for differentiation from the wildtype *S*. Enteritidis strains. SNP analysis confirmed that streptomycin resistance was associated with a Lys43Arg missense mutation in the *rps*L gene whilst 3 missense mutations in *acrB* and 1 missense mutation in *acrA* confer erythromycin sensitivity in AviPro SALMONELLA VAC E. Further mutations Arg242His in *purK* and Gly236Arg in the *hisB* gene were related to adenine and histidine dependencies in Salmovac 440. Unique SNPs were used to construct a database of variants for differentiation of vaccine from the wildtype isolates. Two fragments from each vaccine were represented in the database to ensure high accuracy. Each of the two selected Salmovac 440 fragments differed by 6 SNPs from the wildtype and the AviPro SALMONELLA VAC E fragments differed by 4 and 6 SNPs, respectively. CD-hit software was applied to cluster similar fragments that produced the best fit output when searched with SRST2. The developed vaccine differentiation method was tested with 1,253 genome samples including field isolates of Salmovac 440 (*n* = 51), field isolates of AviPro SALMONELLA VAC E (*n* = 13), *S*. Gallinarum (*n* = 19), *S*. Pullorum (*n* = 116), *S*. Enteritidis (*n* = 244), *S*. Typhimurium (*n* = 810) and achieved 100% sensitivity and specificity.

## Introduction

*Salmonella* Enteritidis is a leading cause of salmonellosis worldwide (1) and more than 80% of investigated outbreaks in Europe have been associated with the consumption of inadequately cooked eggs or foods containing uncooked eggs (https://doi.org/10.2903/j.efsa.2019.5596) (2). Over the last few decades the Colindale phage typing (PT) scheme has played a central role in epidemiological studies of *S*. Enteritidis while there are limitations regarding Pulsed-field Gel Electrophoresis (PFGE) and Multilocus Variable-Number Tandem Repeat Analysis (MLVA) methods and “neither PFGE nor MLVA could distinguish all of the *S*. Enteritidis PT30 from various sources,” for example (3–5). Recent phylogenetic studies based on whole genome sequencing (WGS) have revealed the presence of two separate clonal lineages of *S*. Enteritidis (6, 7). Phage types that dominated in western Europe and Asia, including PT1, PT4, and PT21 occurred in clonal lineage I, while PTs that were most common in North America (i.e., PT8, PT13a, and PT13) comprised the majority of clonal lineage II (6).

Vaccination has been proven to be one of the most successful measures to reduce *Salmonella* Enteritidis infections in poultry farms (8) as it can decrease colonization of the reproductive organs and intestinal tract of laying hens by *Salmonella*, thus reducing egg contamination (9). The prevalence of *S*. Enteritidis in large-scale laying hen holdings may be reduced by 88 percent by vaccination (www.efsa.europa.eu/EFSA/efsa_locale-1178620753812_1178620761896.htm). Live attenuated vaccines have been proposed to provide better protection and are better suited for mass vaccination than inactivated (killed) vaccines (10), although there can be practical problems with effective administration in the field (11). There are two commercially available *S*. Enteritidis live-vaccines in UK; Salmovac 440 (Gallivac SE) (Merial Animal Health Ltd, Lyon, France) and AviPro SALMONELLA VAC E (Lohmann Animal Health GmbH Heinz, Germany). Salmovac 440 vaccine strain has been derived through chemical mutagenesis from *S*. Enteritidis PT 4 that lacks the serovar specific plasmid (https://www.efsa.europa.eu/en/efsajournal/pub/114) and has no antimicrobial resistance genes but contains point mutations resulting in auxotrophism for histidine and adenine. *S*. Enteritidis field isolates are differentiated from the vaccine strain by growing on minimal media with and without histidine and adenine (https://assets.publishing.service.gov.uk/government/uploads/system/uploads/attachment_data/file/551476/pub-salm15-intro.pdf). AviPro SALMONELLA VAC E is a metabolic drift mutant strain derived by chemical mutagenesis from *S*. Enteritidis PT4 (http://www.baltivet.com/en/products/veterinary-products/avipro-salmonella-vac-e/). It has been selected according to attenuation criteria such as prolonged generation time, su*per-sen*sitivity to quinolones and increased permeability of the bacterial membrane. It is capable of surviving long enough inside the bird to stimulate immunity, if administered properly, but incapable of surviving in the environment. Sensitivity to erythromycin and resistance to rifampicin are tested to distinguish the vaccine from *Salmonella* field isolates. It is also highly resistant to streptomycin (https://assets.publishing.service.gov.uk/government/uploads/system/uploads/attachment_data/file/551476/pub-salm15-intro.pdf).

Whole genome sequencing (WGS) has been applied as an epidemiological tool for outbreak investigations as it provides high resolution for comparing genomes (12). In the United Kingdom, where human isolates of *Salmonella* are routinely sequenced, WGS has been used successfully to identify and investigate *Salmonella* outbreaks (13–15). Typing of all *Salmonella* isolates with WGS is planned at Animal and Plant Health Agency (APHA) in a near future and will include differentiation of the vaccine from the field isolates, removing the need for phenotypic testing of vaccine types. Also, phenotypic methods can be time- consuming and subject to some variability, requiring confirmation of colony purity and repeat testing of a proportion of isolates. The national control programs for *Salmonella* in chickens introduced in UK in 2007, 2008, and 2009 for breeders, layers, and broilers, respectively, seek to reduce or maintain low *Salmonella* levels of specified serotypes to targets set out in EU regulations (https://assets.publishing.service.gov.uk/government/uploads/system/uploads/attachment_data/file/183065/salmonella-breeders.pdf). Differentiation of vaccine from field *S*. Enteritidis isolates is essential for monitoring of *S*. Enteritidis in poultry and for targeted disease control measures. As a component of the proposed APHA *Salmonella* WGS typing scheme, we characterized the 2 live *S*. Enteritidis vaccines: Salmovac 440 and AviPro SALMONELLA VAC E used in UK poultry by comparing them with wildtype *S*. Enteritidis using WGS-based approaches. Here we describe the method developed to differentiate the vaccine from field isolates based on SNP differences.

## Materials and Methods

### Bacterial Isolates

The vaccine (Salmovac 440, *n* = 5; AviPro SALMONELLA VAC E, *n* = 5) and the S. Enteritidis wildtype (*n* = 6) strains used for method development were from the APHA *Salmonella* Archives (Addlestone, UK) (Table 1). All strains were kept at −80°C in 1% (w/v) proteose peptone water containing 10% (v/v) glycerol. The methods supplied by the manufacturers for differentiation with *S*. Enteritidis wildtype field isolates, based on growth in minimal media without adding histidine and adenine for Salmovac 440 or in media containing rifampicin, streptomycin and erythromycin to distinguish AviPro SALMONELLA VAC E, were used to confirm the vaccinal identity of isolates (16). A further 1,237 isolates, including field isolates of Salmovac 440 (*n* = 46), field isolates of AviPro SALMONELLA VAC E (*n* = 8), *S*. Gallinarum (*n* = 19), *S*. Pullorum (*n* = 116), *S*. Enteritidis (*n* = 238) and *S*. Typhimurium (*n* = 810) were used to test the developed SNP (single nucleotide polymorphism) differentiation method. All the samples in the study were collected from the environment, such as chicken feces. Therefore, there was no need for the APHA ethics committee to approve the study.

**Table 1:** Sequenced *S*. Enteritidis strains used in this study.

**Id**	**Strain**	**Phage type**
S02105-11	Wildtype	PT9b
S00940-12	Wildtype	PT9b
L00397-09	Wildtype	PT9b
L00453-12	Wildtype	PT9b
FieldSE	Wildtype	PT4
S00668-06	Wildtype	PT4
Strain P125109	Wildtype	PT4
Salmovac 440	Salmovac 440	PT4
S01708-17	Field Isolate of Salmovac 440	PT4
S01805-17	Field Isolate of Salmovac 440	PT4
S01806-17	Field Isolate of Salmovac 440	PT4
S04022-12	Field Isolate of Salmovac 440	PT4
Avipro Vac E	Avipro Vac E	PT4
S03385-15	Field Isolate of Avipro Vac E	PT4
S03815-15	Field Isolate of Avipro Vac E	PT4
S04327-15	Field Isolate of Avipro Vac E	PT4
S04329-15	Field Isolate of Avipro Vac E	PT4

*The gbk file of S. Enteritidis strain P125109 (accession AM933172) was used as a reference strain*.

### Whole-Genome Sequencing and Analysis

Overnight bacterial isolates were collected by centrifugation and resuspended in 0.5 mL 0.1 M PBS (pH 7.2) solution. Genomic DNA was purified with the ArchivePure DNA Cell/Tissue (1 g) kit (5 Prime, Gaithersburg, USA). Purified genomic DNA was fragmented, tagged using the Nextera XT DNA Sample Preparation Kit (Illumina UK) and sequenced at the APHA on the Illumina MiSeq platform based on the manufacturer's instructions. Phylogenetic analysis was carried out with Snippy (https://github.com/tseemann/snippy) to identify all SNPs. Tree of life (iTol) was used to produce midpoint rooted trees (17). To identify differential features that could separate the *S*. Enteritidis wildtype from either one of the two vaccine strains, the draft genomes of *S*. Enteritidis field isolates, Salmovac 440 and AviPro SALMONELLA VacE live vaccine strains as well as 4 field isolates of each of the vaccine strains (Table 1) were analyzed to identify plasmids, plasmid replicons, virulence genes, antimicrobial resistant genes as well as point mutations. SRST2 (18) database searches were carried out to identify plasmids with PlasmidFinder, replicons with PlasmidReplicon database (https://github.com/katholt/srst2), virulence factors with Virulence Factor database (http://www.mgc.ac.cn/VFs/main.htm) and a vaccine database (this study). Antimicrobial resistance genes were identified by Ariba (19) with the Card database (20). The gene presence and absence tables were generated through genome annotation by Prokka (21) after the fastq data were assembled to fasta files with Spades (22) and summarized by Roary (23). Student's *t*-test was carried out compare data between the wildtype and vaccine strains (two tailed distribution for two-sample populations with unequal variance) with Excel spreadsheet function (Microsoft). Blastx was performed with isolate fasta files from Spades assemblies after a protein sequence database was made (24).

## Results

### Comparison of Vaccine and Wild Type *S*. Enteritidis Genomes

Comparative genomic analyses of *S*. Enteritidis field isolates, Salmovac 440 and AviPro SALMONELLA VacE live vaccine strains as well as four field isolates of each vaccine strain idendified by standard laboratory methods were carried out in order to identify genomic features that could differentiate the strains. The searches and identified features are summarized in Table 2. SRST2 plasmid searches identified presence of a *Salmonella* Paratyphi C strain RKS4594 plasmid pSPCV (plasmid CP000858) in the wild type *S*. Enteritidis genomes, the vaccine strain SALMONELLA Vac E and field isolates. However, the 262 bp fragment in PlasmidFinder that identifies the 55,414 base pairs plasmid CP000858 is identical with the 262 bp fragment that identifies the 59,372 bp of *S*. Enteritidis strain-specific plasmid pSEN (HG970000) or pSENV (JN885080) which is present in most *S*. Enteritidis wild type isolates. As expected, the presence of the pSENV plasmid and associated plasmid replicon was confirmed in all wild type and the SALMONELLA Vac E vaccine strain and field isolates but not in the genome of the vaccine strain Salmovac 440, one of the features of this vaccine strain (Rows 1–2, Table 2). The analysis of the AMR genes or mutations identified rifampicin resistance related to the *rpoB* gene in Avipro SALMONELLA Vac E strains (Row 3, Table 2).

**Table 2:** Identification of differential features between groups of the wildtype, Salmovac 440, and Avipro SALMONELLA Vac E from the search results of PlasmidFinder.

		**Wildtype**						**Salmovac 440**					**Avipro Vac E**				
		**L00397-09**	**S2105_11**	**S940_12**	**L00453-12**	**FieldSE**	**S00668-06**	**S04022-12**	**S01708-17**	**S01805-17**	**S01806-17**	**Salmovac**	**S03815-15**	**S04327-15**	**S04329-15**	**S03385-15**	**AviproE**
1	FIIS	1	1	1	1	1	1	0	0	0	0	0	1	1	1	1	1
2	FII_repA	1	1	1	1	1	1	0	0	0	0	0	1	1	1	1	1
3	*rpoB* (SEN3937)	0	0	0	0	0	0	0	0	0	0	0	1	1	1	1	1
4	*pefB* (pSENV_028)	1	1	1	1	1	1	0	0	0	0	0	1	1	1	1	1
5	*pefC* (pSENV_030)	1	1	1	1	1	1	0	0	0	0	0	1	1	1	1	1
6	*pefD* (pSENV_031)	1	1	1	1	1	1	0	0	0	0	0	1	1	1	1	1
7	*rck* (pSENV_039)	1	1	1	1	1	1	0	0	0	0	0	1	1	1	1	1
8	*spvA* (pSENV_002)	1	1	1	1	1	1	0	0	0	0	0	1	1	1	1	1
9	*spvB* (pSENV_003)	1	1	1	1	1	1	0	0	0	0	0	1	1	1	1	1
10	*spvC* (pSENV_004)	1	1	1	1	1	1	0	0	0	0	0	1	1	1	1	1
11	*spvD* (pSENV_005)	1	1	1	1	1	1	0	0	0	0	0	1	1	1	1	1
12	spvR (pSENV_001)	1	1	1	1	1	1	0	0	0	0	0	1	1	1	1	1
13	*envR* (SEN1329)	1	1	1	1	1	1	1	1	1	1	1	0	0	0	0	0
14	*gmr_2* (SEN3937)	1	1	1	1	1	1	1	1	1	1	1	0	0	0	0	0
15	group_503	1	1	1	1	1	1	1	1	1	1	1	0	0	0	0	0
16	group_343	0	0	0	0	0	0	0	0	0	0	0	1	1	1	1	1
17	group_413	0	0	0	0	0	0	0	0	0	0	0	1	1	1	1	1

Row1, plasmid searches with PlasmidFinder searches; Row 2, Plasmid Replicon searches; Row 3, Ariba Card point mutation searches; Rows 4–12, Virulence factor searches; Rows13–17, gene presence and absence searches with Prokka/Roary. Search results: 1, presence; 0, absence. SEN****, locus tag of AM933172; pSENV_***, locus tag of JN885080; Group_***, hypothetical protein.

Furthermore, the 9 pSENV plasmid-associated virulence genes identified in the wildtype and Avipro SALMONELLA Vac E vaccine and field isolates were not present in the Salmovac 440 strains (Rows 4–12, Table 2), confirming the absence of the plasmid in these strains.

The comparison of the annotated wildtype and vaccine genomes revealed the absence of 3 genes: *envR*, a regulator in the *Salmonella* pathogenicity island 2 (SPI2) *gmr_2*, cyclic di-GMP phosphodiesterase and group_503, and a hypothetical protein (Rows 13–15, Table 2) and presence of two hypothetical genes: *group_343* and *group_413* (Rows 16–17) in the vaccine strain Avipro SALMONELLA Vac E strains. A further 62 pSENV plasmid-related genes were absent in Salmovac 440 but present in the wildtype and Avipro SALMONELLA Vac E (Table S1).

### Phylogenetic Analysis With Core SNPs

**Figure 1:**
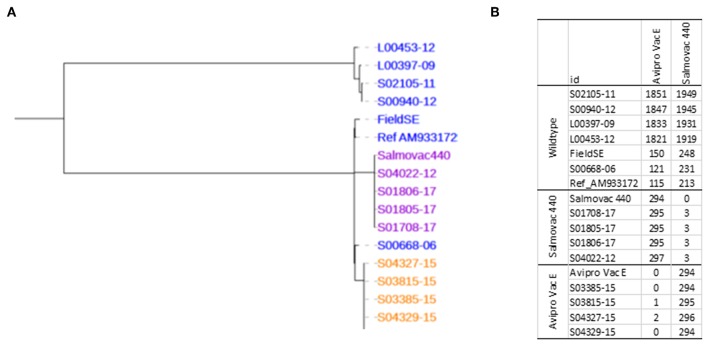
**(A)**, Maximum likelihood tree of *S*. Enteritidis vaccines. Salmovac 440 (purple), AviproVacE= AviPro SALMONELLA VAC E (orange) and the wildtype (blue). **(B)**, SNP distance from AviPro SALMONELLA VAC E and Salmovac 440 to all the samples including the reference (*S*. Enteritidis strain P125109) in the tree.

Phylogenetic analysis of the core SNPs indicated a close relatedness of both vaccine strains with the reference *S*. Enteritidis strain P125109 (AM933172). The distance was 115 SNPs between AviPro SALMONELLA VAC E and the reference and 213 SNPs between Salmovac 440 and the reference (Figure 1). The Salmovac 440 vaccine reference strain and the vaccine strains recovered from farms differed by up to 3 SNPs; while the AviPro SALMONELLA VAC E reference strain differed from the vaccine strains recovered from farms by 0–2 SNPs. All SNPs are listed between the reference and AviPro *Salmonella* VAC E or Salmonvac 440 (Table S2).

### The Use of Point Mutations to Explain Some Properties of Salmovac 440 and AviPro SALMONELLA VAC E

A point mutation in the *rpoB* gene was identified by Ariba Card relating to AviPro SALMONELLA VAC E's rifampicin resistance (Table 2). Ariba Card does not identify the position in the gene, however, we identified a missense mutation, Ser531Phe in the *rpo*B gene (Row 1, Table 3) that could explain the rifampicin resistance of the AviPro SALMONELLA VAC E vaccine strain and a missence mutation Lys43Arg in the *rpsL* gene that could explain the resistance to streptomycin (Row 2, Table 3).

**Table 3:** Identification of unique SNPs linked to vaccine strain properties of antimicrobial resistance and histidine and adenine dependencies.

		**Wildtype**							**Salmovac 440**					**Avipro Vac E**							
	**POS**	**Ref_AM933172**	**FieldSE**	**L00397-09**	**L00453-12**	**S00668-06**	**S02105-11**	**S00940-12**	**Salmovac 440**	**S01708-17**	**S01805-17**	**S01806-17**	**S04022-12**	**Avipro Vac E**	**S03385-15**	**S03815-15**	**S04327-15**	**S04329-15**	**GENE**	**PRODUCT**	**EFFECT**
1	4239703	C	C	C	C	C	C	C	C	C	C	C	C	T	T	T	T	T	*rpoB*	DNA-directed RNA polymerase beta-subunit	Ser531Phe
2	3484198	T	T	T	T	T	T	T	T	T	T	T	T	C	C	C	C	C	*rpsL*	30S ribosomal subunit protein S12	Lys43Arg
3	509506	C	C	C	C	C	C	C	C	C	C	C	C	T	T	T	T	T	*acrB*	acriflavin resistance protein B	Gly755Asp
4	510524	C	C	C	C	C	C	C	C	C	C	C	C	T	T	T	T	T	*acrB*	acriflavin resistance protein B	Val416Ile
5	510901	C	C	C	C	C	C	C	C	C	C	C	C	T	T	T	T	T	*acrB*	acriflavin resistance protein B	Gly290Asp
6	512087	C	C	C	C	C	C	C	C	C	C	C	C	T	T	T	T	T	*acrA*	acriflavin resistance protein A precursor	Gly300Glu
7	575879	C	C	C	C	C	C	C	T	T	T	T	T	C	C	C	C	C	*purK*	phosphoribosylaminoimidazole carboxylase ATPase subunit	Arg242His
8	2153628	C	C	C	C	C	C	C	T	T	T	T	T	C	C	C	C	C	*hisG*	ATP phosphoribosyltransferase	Leu195Leu
9	2156212	G	G	G	G	G	G	G	A	A	A	A	A	G	G	G	G	G	*hisC*	histidinol-phosphate aminotransferase (imidazole)	Gln288Gln
10	2157130	G	G	G	G	G	G	G	A	A	A	A	A	G	G	G	G	G	*hisB*	bifunctional histidine biosynthesis protein (imidazoleglycerol-phosphate dehydratase; histidinol phosphatase)	Gly236Arg

Furthermore, 3 missense mutations in *acrB* and 1 missense mutation in *acrA* genes found in AviPro SALMONELLA VAC E are likely to be associated with erythromycin sensitivity (Rows 3–6, Table 3).

The adenine dependency of Salmovac 440 could be explained by a missense mutation Arg242His in *purK*, the N5-Carboxyaminoimidazole ribonucleotide (N5-CAIR) synthetase gene of the purine biosynthesis pathway (Row 7, Table 3). Three mutations in the histidine biosynthesis pathway were identified when Salmovac 440 was compared with the wildtype (Rows 8–10, Table 3); the mutation in the *hisB* gene was a missense mutation, Gly236Arg, suggesting that the mutation might give Salmovac 440 the property of histidine dependence.

Phylogenetic analysis considers only single nucleotide changes. To rule out any possibility of insertions or deletions in the pathways of efflux pump, histidine biosynthesis, and purine biosynthesis, Blastx was carried out to find out any amino acid sequence changes. The analyses showed no evidence of insertions or deletions and the mis-sense mutations 1 in *acrA*, 3 in *acrB* unique to AviPro SALMONELLA VAC E and 1 in *hisB* and 1 in *purK* unique to Salmovac 440 identified by phylogenetic analysis, were confirmed (Table 3 and Table S3).

### Attenuation of Salmovac 440 and AviPro SALMONELLA VAC E Vaccine Strains

Salmovac 440 has lost the pathogenic plasmid that encodes a number of virulence factors (Table 2). This may partially explain the attenuation of Salmovac 440. However, for AviPro SALMONELLA VAC E, Prokka/Roary/TTest only identified *env*R as a potential candidate for attenuation (Row 13, Table 2) a regulator in the *Salmonella* pathogenicity island 2 (SPI2). Other possible sources for attenuation were sought, such as point mutations in known genes associated with virulence. Phylogenetic analysis identified a total of 96 SNPs unique to AviPro SALMONELLA VAC E of which 56 were missense SNPs. Among these 56 missense SNPs, 20 were in genes reported to be associated with virulence (Table 4). This group of genes were from diverse functions: 1 in iron uptake (Row 6), 3 in potassium transport (Rows 9–12) and 7 genes related to antimicrobial resistance through point mutations (Rows 1–4 and 15–17).

**Table 4:** Missense SNPs unique to AviPro SALMONELLA VAC E in the genes associated with virulence.

		**Wildtype**							**AviproVacE**								
	**POS**	**Ref_AM933172**	**FieldSE**	**L00397-09**	**L00453-12**	**S00668-06**	**S02105-11**	**S00940-12**	**AviproVacE**	**S03385-15**	**S03815-15**	**S04327-15**	**S04329-15**	**GENE**	**PRODUCT**	**EFFECT**	**REFERENCES**
1	512087	C	C	C	C	C	C	C	T	T	T	T	T	*acrA*	acriflavin resistance protein A precursor	Gly300Glu	(25, 26)
2	509506	C	C	C	C	C	C	C	T	T	T	T	T	*acrB*	acriflavin resistance protein B	Gly755Asp	(25, 26)
3	510524	C	C	C	C	C	C	C	T	T	T	T	T	*acrB*	acriflavin resistance protein B	Val416Ile	(25, 26)
4	510901	C	C	C	C	C	C	C	T	T	T	T	T	*acrB*	acriflavin resistance protein B	Gly290Asp	(25, 26)
5	770691	C	C	C	C	C	C	C	T	T	T	T	T	*cydB*	Cytochrome d ubiquinol oxidase subunit II	Thr66Ile	(27, 28)
6	614496	C	C	C	C	C	C	C	T	T	T	T	T	*fepA*	ferrienterobactin receptor precursor	Ala396Thr	(29)
7	393597	C	C	C	C	C	C	C	T	T	T	T	T	*foxA*	ferrioxamine B receptor precursor	Thr543Ile	(30, 31)
8	456168	C	C	C	C	C	C	C	T	T	T	T	T	*ispA*	Geranyltranstransferase	Arg210His	(32)
9	744792	C	C	C	C	C	C	C	T	T	T	T	T	*kdpA*	potassium-transporting ATPase A chain	Asp439Asn	(33)
10	744996	C	C	C	C	C	C	C	T	T	T	T	T	*kdpA*	potassium-transporting ATPase A chain	Val371Met	(33)
11	745001	C	C	C	C	C	C	C	T	T	T	T	T	*kdpA*	potassium-transporting ATPase A chain	Gly369Asp	(33)
12	739356	C	C	C	C	C	C	C	T	T	T	T	T	*kdpD*	sensor protein KdpD	Val805Ile	(33, 34)
13	805115	C	C	C	C	C	C	C	T	T	T	T	T	*modA*	molybdate-binding periplasmic protein precursor	Thr98Ile	(35)
14	806676	C	C	C	C	C	C	C	T	T	T	T	T	*modC*	molybdenum transport ATP-binding protein ModC	Ser130Phe	(35)
15	4239703	C	C	C	C	C	C	C	T	T	T	T	T	*rpoB*	DNA-directed RNA polymerase beta-subunit	Ser531Phe	(36)
16	2950458	A	A	A	A	A	A	A	G	G	G	G	G	*rpoS*	RNA polymerase sigma subunit RpoS (sigma-38)	Leu263Ser	(37, 38)
17	3484198	T	T	T	T	T	T	T	C	C	C	C	C	*rpsL*	30S ribosomal subunit protein S12	Lys43Arg	(39–41)
18	1391886	T	T	T	T	T	T	T	C	C	C	C	C	*trpA*	Tryptophan synthase alpha chain	Tyr175Cys	(42)
19	865528	C	C	C	C	C	C	C	T	T	T	T	T	*ybiT*	ABC transporter ATP-binding protein	Pro465Ser	(43)
20	4047472	C	C	C	C	C	C	C	T	T	T	T	T	*yigP*	Conserved hypothetical protein	Thr193Ile	(44)

### WGS Based Differentiation Between Vaccine and the Wild Type Strains

To construct vaccine differentiation database, total SNPs were identified from the phylogenetic analysis of the 16 sequenced genomes (Table 1) using the *S*. Enteritidis strain P125109 (accession AM933172) as reference. Unique SNPs to Salmovac 440 were identified after comparison with the wildtype *S*. Enteritidis and AviPro SALMONELLA VAC E genomes. Two regions were selected to represent each vaccine strain, Salmovac 440 and AviPro SALMONELLA VAC E, and pair wise fragments were created so that one represented Salmovac 440 and the other the wildtype. The paired fragments were identical except for 6 SNPs in each region (Figure S1). CD-hit (45) was performed to cluster the paired fragments together so that when the fragments in the database are used as references for SRST2 (18) only one fragment from a pair gets reported: either a vaccine fragment or a wildtype fragment but not both.

The SNPs unique to Salmovac 440 were located in two genomic regions, region 831G from position 2145373 to 2157130 of the reference AM933172 with 6 different nucleotides in positions: 2145373 (WT, G; 831G, A), 2148431 (WT, G; 831G, A), 2150004 (WT, G; 831G, A), 2153628 (WT, C; 831G, T), 2156212 (WT, G; 831G, A), and 2157130 (WT, G; 831G, A) and region 832G from position 854486 to 863710 with 6 different nucleotides in positions: 854486 (WT, C; 832G, T), 855295 (WT, C; 832G, T), 856153 (WT, C; 832G, T), 858894 (WT, C; 832G, T), 863334 (WT, C; 832G, T), 863710 (WT, C; 832G, T) (Figure S1). The two regions for the AviPro SALMONELLA VAC E strain were 509256 to 512337 of the reference AM933172 with 4 SNPs differences in positions: 509506 (WT, C; 8321, T), 510524 (WT, C; 8321, T), 510901 (WT, C; 8321, T), 512087 (WT, C; 8321, T), and Avipro Vac E 8322 from position 802121 to 806926 with 6 SNPs differences in positions: 802371 (WT, C; 8322, T), 802460 (WT, C; 8322, T), 803918 (WT, C; 8322, T), 805115 (WT, C; 8322, T), 805353 (WT, C; 8322, T), 806676 (WT, C; 8322, T) (Figure S1). The identified genomic regions were used to create pair wise fragments, one fragment representing the vaccine strain and the second representing the wild type, differing only by the identified SNPs.

The sensitivity and specificity of the vaccine database of variants in distinguishing field vaccine strains from wild type strains was 100%. The database was tested with a total of 1,253 sequenced genomes including Salmovac 440 (*n* = 51) and AviPro SALMONELLA VAC E (*n* = 13) field isolates, *S*. Gallinarum (*n* = 19), *S*. Pullorum (*n* = 116), wildtype *S*. Enteritidis (*n* = 244), and *S*. Typhimurium (*n* = 810) (Table 5).

**Table 5:** The sensitivity and specificity of the fragments in the database.

**Fragments**	**SE Gallivac (*n* = 51)**	**SE Avipro Vac E (*n* = 13)**	**ST Avipro Vac T (*n* = 12)**	**ST Salmoporc STM (*n* = 12)**	***S*. Gallinarum (*n* = 19)**	***S*. Pullorum (*n* = 116)**	***S*. Enteritidis (*n* =2 44)**	***S*. Typhimurium (*n* = 810)**	**Sensitivity %**	**Specificity %**
831G_Gallivac	51	0	0	0	0	0	0	0	100	100
832G_Gallivac	51	0	0	0	0	0	0	0	100	100
8321_AviproE	0	13	0	0	0	0	0	0	100	100
8322_AviproE	0	13	0	0	0	0	0	0	100	100

*The vaccine strains were farm isolates from fecal, post-mortem or environmental samples identified by standard laboratory methods. AviproVacE, Avipro SALMONELLA VAC E*.

## Discussion

In this study, using whole sequencing approaches, we characterized some of the genomic properties of the vaccine strains Salmovac 440 and AviPro SALMONELLA VAC E. Deletion of the *tolC, acrB* or *acrAB* genes is linked to strains with increased susceptibility to antimicrobials, including erythromycin (46). In AviPro SALMONELLA VAC E, phylogenetic analysis identified 3 missense mutations in *acrB* and 1 missense mutation in *acrA* (Rows 3–6, Table 3). In *E. coli*, multiple mutations in the *acrB* gene increase the susceptibility to erythromycin while individually they do not result in any changes of sensitivity, and the mutation in strain T37W even increases resistance to antimicrobials (47). Therefore, the sensitivity in AviPro SALMONELLA VAC E may be due to the possibility that these 4 mutations in *acrAB* have changed some properties of AcrAB efflux pump. The *strA-strB* genes are most likely associated with high levels of streptomycin resistance, whereas the *aadA* gene confers low-level resistance (48). Streptomycin resistance in *M*. *tuberculosis* isolates is frequently linked to missense mutations in the *rpsL* gene for ribosomal protein S12 or in the *rrs* gene for nucleotide substitutions in the 16S rRNA gene (49). In this study, we detected a missense mutation, Lys43Arg, in the *rpsL* gene (Table 3). The same amino acid replacement Lys43Arg of *rpsL* has been described in *Mycobacterium tuberculosis* and *Helicobacter pylori* (50, 51); while in *E. coli* the change is Lys42Arg (52). A missence point mutation Ser531Phe in the *rpoB* gene we detected in this study is most likely linked to the rifampicin resistance of SALMONELLA VAC E strain. Several mutations in the *rpoB* gene have been described to reduce the susceptibility to rifampicin in clinical *Mycobacterium tuberculosis* isolates that contribute to various degree of fitness cost to the strain (53).

HisB is bifunctional since the C-terminal domain catalyzes as IGP dehydratase (the sixth step) while the N-terminal domain as Hol-P phosphatase (the eighth step) in the histidine biosynthesis pathway (54). A total of 1,020 independent histidine-requiring mutations were isolated in the histidine operon after strain LT2 of *S*. Typhimurium was treated with N-methy1-N'-nitro-N-nitrosoguanidine, the same agent used for Salmovac 440 mutagenesis; many of these mutations were found in the *hisB* gene (55).

Salmovac 440 is lacking the pathogenic plasmid pSENV that encodes a number of virulence factors (Table 2). The plasmid could be associated with much of the virulence as the *spv* (*Salmonella* plasmid virulence) is considered crucial for the phenotype of *S*. Enteritidis (56) and it has been shown that *spvB* mutants are avirulent in mice (57). Histidine-requiring mutations have also been shown to lead to attenuation. In *Aspergillus fumigatus*, mutation in *hisB* causes histidine auxotrophy and attenuation of virulence in 3 murine models: pulmonary infection, systemic infection, corneal infection, and in the wax moth larvae model (58). In *Xanthomonas oryzae* pv. Oryzicola which triggers bacterial leaf streak in rice, two genes in histidine biosynthesis operon, *trpR* and *hisB*, were identified to be essential for virulence and bacterial growth in plants (59). Adenine is one of the products of purine biosynthesis. Disrupted *de novo* purine biosynthesis has been revealed to attenuate the virulence of several pathogens, such as *Salmonella, Burkholderia, Brucella*, and *Francisella* (60–64). Therefore, the attenuation of Salmovac 440 is most likely the result of the combination of absence of the virulence plasmid and histidine and adenine dependencies.

As for AviPro SALMONELLA VAC E, the attenuation may also be the result of multiple factors: e.g., the missing *envR* gene (Table 2) and point mutations in 20 virulence-associated genes (Table 4). *Env*R as a potential candidate for attenuation (Row 13, Table 2) is a regulator in the *Salmonella* pathogenicity island 2 (SPI2) that encodes type III secretion system (T3SS) that changes the host cell functions and facilitate intracellular replication (65, 66). The RpoB H526D mutant (Rif) displayed reduced survival compared with control strains in *Mycobacterium tuberculosis* (36). Mutations in *rpsL* that result in streptomycin resistance indicated that the K43N and K43T mutations were pleiotropic, showing reduced virulence in *Erwinia carotovora* (39). Attenuation of an avian pathogenic *Escherichia coli* strain resulted from a point mutation in *rpsL* (40). Direct evidence of point mutations leading to attenuation is also observed in *Salmonella* Typhimurium mutants resistant to streptomycin or rifampicin that become avirulent in mice (41).

With the wide use of *S*. Enteritidis live vaccines on chicken farms, a reliable and rapid differentiation method is essential. Currently, there are two methods in use. One is phenotypic typing based on manufacturers' instructions. For AviPro SALMONELLA VAC E, the vaccine strain is rifampicin resistant and erythromycin sensitive; while for Salmovac 440, the vaccine strain requires histidine and adenine supplements in order to grow on minimal media. The second method uses TaqMan-qPCR to differentiate Salmovac 440 and AviPro SALMONELLA VAC E vaccine strains from the wildtype Enteritidis (67). To produce this test, the authors sequenced the whole genomes of both vaccine strains and identified SNPs that were used to design PCR probes based on 2 SNPs for AviPro SALMONELLA VAC E and 1 SNP for Salmovac 440. The real time PCR method identified all 30 Salmovac 440 and 7 AviPro SALMONELLA VAC E vaccine strains (100% sensitivity) and eliminated all of the 97 wild type *S*. Enteritidis as well as other *S*. *enterica* strains (100% specificity). The method we developed in this study was also based on SNP differences between the vaccine strains and the wildtype however, the short regions described in Maurischat et al. (67) used in real tim PCR differentiation, 146 bp in *nhaA* for Salmovac SE and 88 bp in *kdpA* for AviPro SALMONELLA VAC E, were not sufficient for alignment with Illumina short reads with high coverage. To ensure high sensitivity and specificity we selected two regions each to represent the vaccine strains and more SNPs than the PCR method (67) (Figure S1). In the real time PCR differentiation study, the authors tested the specificity with non-*Salmonella* species as well as *Salmonella* serovars (67); while in this study, we aimed to differentiate vaccine strains from genomes of isolates typed as *Salmonella* Enteritidis, Typhimurium, Gallinarum or Pullorum by our WGS serotyping pipeline. Further database development will include an additional 3 live vaccines: AviPro SALMONELLA VAC T, Salmoporc STM and Nobilis SG 9R (Tang et al. unpublished data). Although the differentiation using the developed vaccine database showed 100% sensitivity and 100% specificity (Table 5), we have also developed a scheme to ensure typing accuracy in case of mixed results. If a sample is typed as wildtype in one region and vaccine in another, this sample will be analyzed using phylogeny based on a panel of isolates shown in Figure 1, including wildtype *S*. Enteritidis isolates and both Salmovac 440 and AviPro SALMONELLA VAC E vaccines so that all SNPs will be considered. With the mean mutation rate across all *S*. Enteritidis lineages being 2.2 × 10–7 substitutions per site per year or 1.01 SNPs per genome per year (68) both attenuation of the vaccine strains and SNPs used for differentiation should be stable.

In conclusion, we characterized Salmovac 440 and AviPro SALMONELLA VAC E vaccine strains and identified genomic features that could have resulted in attenuation, resistance to rifampicin and streptomycin in AviPro SALMONELLA VAC E and histidine and adenine dependencies in Salmovac 440. We developed a database of highly specific SNP variants that could differentiate vaccine from wild type strains with 100% sensitivity and 100% specificity. The knowledge and methods from this study could be applied for characterization and differentiation of other *Salmonella* vaccine strains that are in use outside UK.

## Data Availability Statement

The whole genome sequencing fastq files for this study can be found in PRJEB33366 of the European Nucleotide Archive (https://www.ebi.ac.uk/ena).

## Author Contributions

LP, YT, and RD designed the study. YT and LP analyzed the data and drafted the manuscript. All authors read through and approved the manuscript.

### Conflict of Interest

The authors declare that the research was conducted in the absence of any commercial or financial relationships that could be construed as a potential conflict of interest.
