# Supplementary material for: Identification of Genetic Features for Attenuation of Two Salmonella Enteritidis Vaccine Strains and Differentiation of These From Wildtype Isolates Using Whole Genome Sequencing
Source: Front Vet Sci. 2019 Dec 18;6:447. doi: 10.3389/fvets.2019.00447 (PMC6930191; doi:10.3389/fvets.2019.00447)
Supplement: Supplementary file 4 [file Image_1.pdf]

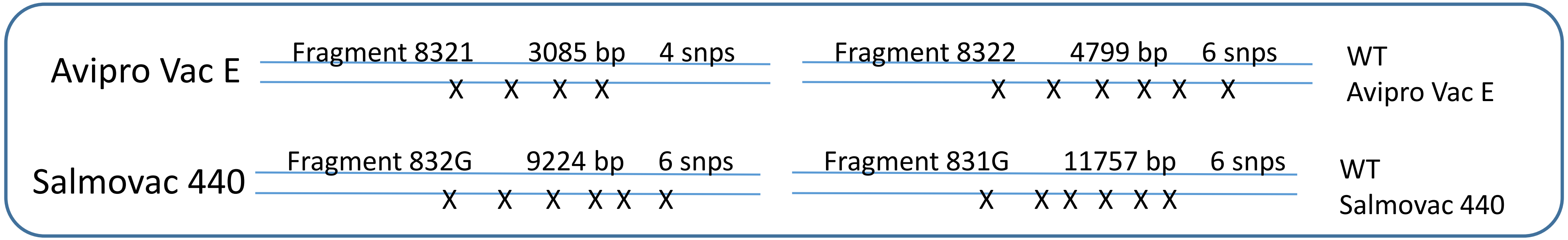

Figure S1. An S. Enteritidis vaccine differentiation database for SRST2. The pair wise fragments were clustered together with CD-hit so that SRST2 only reports the best aligned fragments.
